# Highly stable and efficient all-inorganic lead-free perovskite solar cells with native-oxide passivation

**DOI:** 10.1038/s41467-018-07951-y

**Published:** 2019-01-03

**Authors:** Min Chen, Ming-Gang Ju, Hector F. Garces, Alexander D. Carl, Luis K. Ono, Zafer Hawash, Yi Zhang, Tianyi Shen, Yabing Qi, Ronald L. Grimm, Domenico Pacifici, Xiao Cheng Zeng, Yuanyuan Zhou, Nitin P. Padture

**Affiliations:** 1 School of Engineering, Brown University, Providence, Rhode Island 02912 USA; 2 Department of Chemistry, University of Nebraska-Lincoln, Lincoln, Nebraska 68588 USA; 3 Department of Chemistry and Biochemistry, Worcester Polytechnic Institute, Worcester, MA 01609 USA; 4 Energy Materials and Surface Sciences Unit, Okinawa Institute of Science and Technology Graduate University, Okinawa, 904-0495 Japan

## Abstract

There has been an urgent need to eliminate toxic lead from the prevailing halide perovskite solar cells (PSCs), but the current lead-free PSCs are still plagued with the critical issues of low efficiency and poor stability. This is primarily due to their inadequate photovoltaic properties and chemical stability. Herein we demonstrate the use of the lead-free, all-inorganic cesium tin-germanium triiodide (CsSn_0.5_Ge_0.5_I_3_) solid-solution perovskite as the light absorber in PSCs, delivering promising efficiency of up to 7.11%. More importantly, these PSCs show very high stability, with less than 10% decay in efficiency after 500 h of continuous operation in N_2_ atmosphere under one-sun illumination. The key to this striking performance of these PSCs is the formation of a full-coverage, stable native-oxide layer, which fully encapsulates and passivates the perovskite surfaces. The native-oxide passivation approach reported here represents an alternate avenue for boosting the efficiency and stability of lead-free PSCs.

## Abstract (web summary)

Replacing the toxic lead in the state-of-the-art halide perovskite solar cells is highly desired but the device performance and stability are usually compromised. Here Chen et al. develop inorganic cesium tin and germanium mixed-cation perovskites that show high operational stability and efficiency over 7%.

## Introduction

The promise of high efficiency and low cost has been propelling perovskite solar cells (PSCs) research over the past decade or so^1–4^. While the record power conversion efficiency (PCE) of PSCs is now approaching 24%^5^, rivaling that of silicon-based solar cells, the state-of-the-art PSCs employ lead-based organic–inorganic halide perovskite absorber materials. The toxicity of lead associated with the lifecycle of these PSCs is a serious concern, and it may prove to be a major hurdle in the path toward their commercialization^6–9^. Thus, significant effort is being devoted toward the development of low-cost, efficient lead-free PSCs. Several low-toxicity cations have been proposed for replacing Pb(II) in halide perovskites, including Ag(I)^10^, Bi(III)^11,12^, Sb(III)^13^, Ti(IV)^14,15^, Ge(II)^16^, and Sn(II)^17,18^. Among these candidates, halide perovskites based on Sn(II) have shown the highest PCE, and, thus, have attracted the most attention in the PSC field. Typical Sn-based halide perovskites that have been studied include CH_3_NH_3_SnI_3_ (MASnI_3_), HC(NH_2_)_2_SnI_3_ (FASnI_3_), and CsSnI_3_. While PSCs based on MASnI_3_ and FASnI_3_ perovskites have been shown to deliver high PCE, up to 9%^19^, these materials have intrinsically low stability^20,21^. This is primarily attributed to the presence of the organic cation, which is prone to facile volatilization. In this context, the all-inorganic lead-free CsSnI_3_ perovskite becomes a more attractive candidate^17,22^. However, the facile oxidation of Sn(II) to Sn(IV), and attendant phase instability in the CsSnI_3_ perovskite, results in the rapid degradation of its properties^23^. The most effective strategy that has been proposed for mitigating this issue is to incorporate Sn(II)-halide additive (SnF_2_^24^, SnCl_2_^25^, and SnI_2_^26^), but the resulting PSCs show maximum PCE of only 4.81%, and no operational stability data on these PSCs has been reported. This calls for new stabilization approaches that can boost the stability and PCE of CsSnI_3_-based PSCs simultaneously.

Herein, we report the surprising discovery that by simply alloying Ge(II) in CsSnI_3_ to form a CsSn_0.5_Ge_0.5_I_3_ composition perovskite, its thin films can become highly stable and air-tolerant. While the favorable Goldschmidt tolerance (0.94) and octahedral (0.4) factors in CsSn_0.5_Ge_0.5_I_3_ contribute to the structural stability of this alloy (Supplementary Fig. 1), the extremely high oxidation activity of Ge(II) enables the rapid formation of an ultrathin (<5 nm) uniform native-oxide surface passivating layer on the CsSn_0.5_Ge_0.5_I_3_ perovskite, imparting it with superior stability, even compared with the prototypical MAPbI_3_ perovskite. We also demonstrate a facile one-step vapor-processing method for the deposition of CsSn_0.5_Ge_0.5_I_3_ perovskite thin films, leading to PSCs with PCE up to 7.11%. We further show that these CsSn_0.5_Ge_0.5_I_3_ PSCs are highly stable upon continuous operation under 1-sun illumination for over 500 h. The extraordinary stability of the CsSn_0.5_Ge_0.5_I_3_ perovskite is explained using coupled experiments and theory.

## Results

### Synthesis and processing of CsSn_0.5_Ge_0.5_I_3_ perovskites

**Fig. 1:**
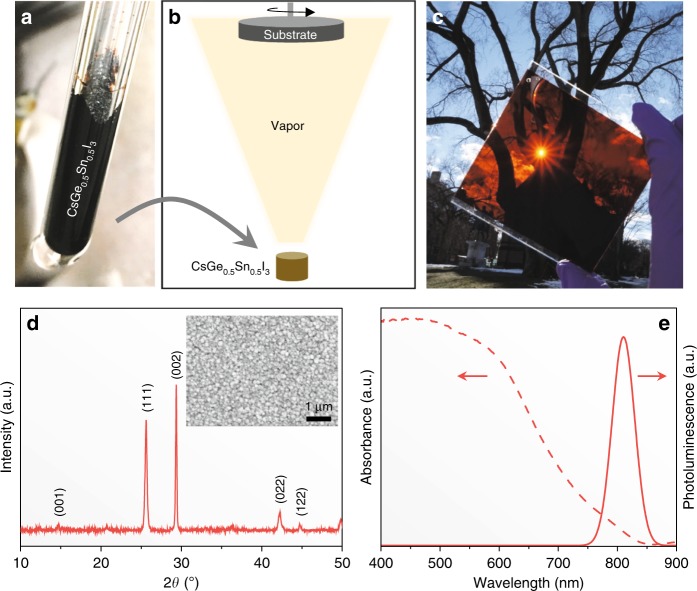
Film synthesis and characterization. **a** Photograph of as-synthesized CsSn_0.5_Ge_0.5_I_3_ perovskite solid using the melt-crystallization method. **b** Schematic illustration of the single-source evaporation method for the deposition of ultrasmooth CsSn_0.5_Ge_0.5_I_3_ perovskite thin film. **c** Photograph of an as-synthesized large-area CsSn_0.5_Ge_0.5_I_3_ perovskite thin film on a glass substrate showing dark reddish color. **d** Indexed XRD pattern of the fresh CsSn_0.5_Ge_0.5_I_3_ perovskite thin film (inset: top-view SEM image of the microstructure). **e** Absorption and steady-state PL spectra of a fresh CsSn_0.5_Ge_0.5_I_3_ perovskite thin film

Figure 1a is a photograph of CsSn_0.5_Ge_0.5_I_3_ perovskite powder synthesized by solid-state reaction (at 450°C) in an evacuated Pyrex glass tube. The indexed X-ray diffraction pattern (XRD) in Supplementary Fig. 1a confirms that this powder is a single-phase perovskite, and it appears to crystallize in the space group of *R3m*. Its comparison with the XRD pattern of reference CsGeI_3_ perovskite powder (space group *R3m*), synthesized using the same procedure, in Supplementary Fig. 1b shows peak shifts to lower 2*θ* angles. This indicates an expansion of the unit cell, as expected with the substitution of the smaller Ge(II) by the larger Sn(II). Reference black phase B-γ-CsSnI_3_ perovskite, which was also synthesized using the same procedure, crystallizes in the orthorhombic (space group *Pnma*) structure; Supplementary Fig. 1c shows its XRD pattern. (More detailed crystallographic analyses of CsSn_1–*x*_Ge_*x*_I_3_ perovskite alloys will be studied in the future to resolve the symmetry transition with the increase of Ge content.)

The CsSn_0.5_Ge_0.5_I_3_ perovskite powder was used to evaporate thin films onto various substrates, as shown schematically in Fig. 1b. Figure 1c is a photograph of such a film deposited on a 10 × 10-cm^2^ glass substrate showing dark reddish color. The XRD pattern in Fig. 1d confirms the single-phase nature of the CsSn_0.5_Ge_0.5_I_3_ perovskite thin film (on a glass substrate). The inset within Fig. 1d shows scanning electron microscope (SEM) image of the top surface. SEM image of the cross section is presented in Supplementary Fig. 2a, where the typical film thickness is about 200 nm, and the grain size is estimated at about 80 nm. The films appear highly uniform, full-coverage, and ultrasmooth, where the root-mean-square roughness is found to be 2.1 nm, as revealed in the atomic force microscope (AFM) image in Supplementary Fig. 2b.

Figure 1e presents optical absorption and steady-state photoluminescence (PL) spectra of the CsSn_0.5_Ge_0.5_I_3_ perovskite thin film. Good absorption is observed across the visible region, with an edge at about 840 nm, although the estimated Urbach energy is relatively high (37 meV), which could be related to the intrinsic Sn vacancies^27,28^. The PL emission peak is centered at about 830 nm, which is consistent with the absorption. The PL peak is relatively sharp (FWHM 52 nm), a hallmark of a good light-absorber material. The PL emission was also mapped over a 50 × 50-μm^2^ area at various locations on the thin films (Supplementary Fig. 3), confirming the optical uniformity across the entire film. The Tauc plot of the thin films in Supplementary Fig. 4a indicates an optical bandgap of about 1.50 eV, which is consistent with that predicted in our earlier computational studies^29^. This bandgap lies in-between the bandgaps of CsSnI_3_ (1.31 eV) and CsGeI_3_ (1.63 eV) perovskites, which is to be expected due to the upshift of the valence band maximum (VBM) with the substitution of Sn(II) for Ge(II) in CsGeI_3_. Supplementary Fig. 4b plots the real and the imaginary parts of the refractive index of the CsSn_0.5_Ge_0.5_I_3_ perovskite thin films as a function of the wavelength.

### Native-oxide surface passivation in CsSn_0.5_Ge_0.5_I_3_ perovskite

**Fig. 2:**
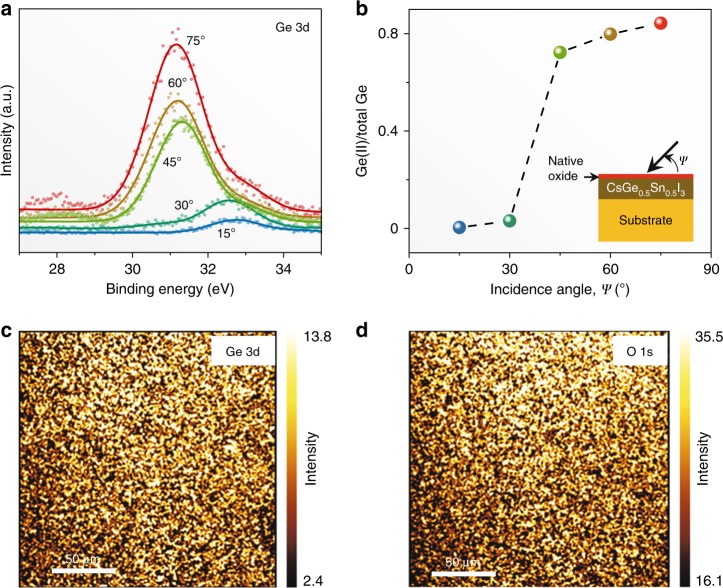
XPS characterization. **a** Ge 3d XPS spectra, at different incidence angles, from CsSn_0.5_Ge_0.5_I_3_ perovskite thin film that has been exposed to air. **b** Corresponding plot of the fraction of Ge(II) vs. the incidence angle. **c**, **d** XPS maps of Ge 3d (33 eV) and O 1s (532 eV), respectively, from the same area of the thin film

As soon as the CsSn_0.5_Ge_0.5_I_3_ perovskite thin films are exposed to air, a stable native-oxide layer forms on the surface, within 30 s. Figure 2a plots Ge 3d X-ray photoelectron spectroscopy (XPS) spectra at different incidence angles. At shallow angles, the presence of Ge(IV) is detected (binding energy 33.0 eV). Since the surface roughness is about 2.1 nm and the native-oxide layer is expected to be less than 5-nm thick, the surface layer is sampled primarily at such shallow angles. With increasing incidence angle, the Ge(II) peak (binding energy 31.0 eV) dominates, as the underlying CsSn_0.5_Ge_0.5_I_3_ perovskite thin film is sampled primarily. These spectra are deconvoluted, and the estimated Ge(II) content (relative to total Ge) is plotted in Fig. 2b. A sharp drop in the Ge(II) content at incidence angles 30 to 45° indicates a distinct native-oxide layer comprising Ge(IV) primarily. XPS maps (normal incidence angle) of Ge 3d (33.0 eV) and O 1s (532.0 eV) in Fig. 2c, d, respectively, of the same surface region show a strong correlation^30,31^, confirming the formation of a Ge(IV)-rich native oxide. Supplementary Fig. 5a plots Sn 3d XPS spectra as a function of incidence angle. Since the binding energies for Sn(II) (486.0 eV) and Sn(IV) (486.6 eV) are very close, it is difficult to discern the oxidation state of Sn. Nevertheless, the Sn:Ge atomic ratio is extracted from the data in Supplementary Fig. 5a and Fig. 2a, and it is plotted in Supplementary Fig. 5b as a function of incidence angle. It can be seen that there is a small amount (<10%) of Sn present in the native oxide. In a related experiment, Ar sputtering (15 s) was utilized in situ to remove the native-oxide layer at the surface. The XPS results (15° incidence angle) after Ar sputtering (Supplementary Figs. 6a and 6b) show Ge(II)-rich and O-lean surface corresponding to the CsSn_0.5_Ge_0.5_I_3_ perovskite, which confirms that the thickness of the oxide layer is within 5 nm. Taken together, the XPS results indicate that a uniform layer of Sn-containing Ge(IV)-rich native oxide (<5 nm) forms on the surface of the CsSn_0.5_Ge_0.5_I_3_ perovskite thin films. As with most native-oxide layers that are less than 5 nm in thickness, this native-oxide layer is expected to be amorphous.

### Native oxide passivated CsSn_0.5_Ge_0.5_I_3_ perovskite stability

**Fig. 3:**
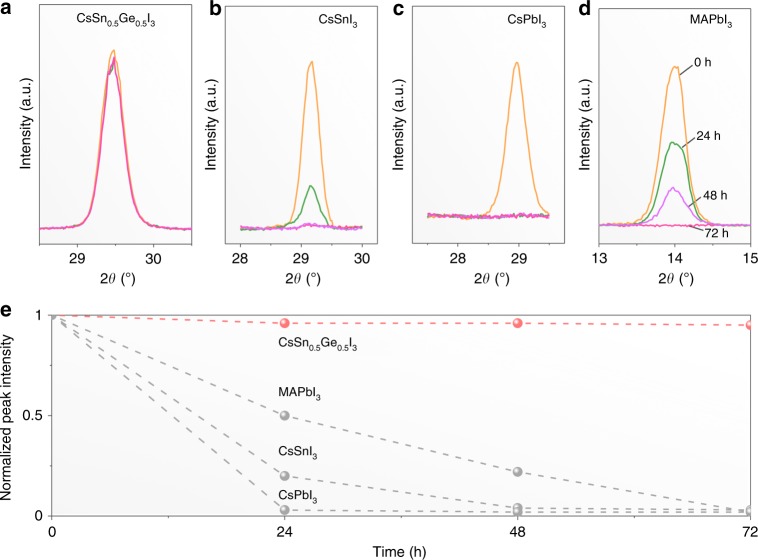
Thin-film stability. XRD patterns of perovskite thin films before and after exposure for 24, 48, and 72 h to light-soaking (1 sun) at approximately 45 ˚C and 80% RH: **a** CsSn_0.5_Ge_0.5_I_3_, **b** CsSnI_3_, **c** CsPbI_3_, and **d** MAPbI_3_. **e** Plots of relative XRD peak intensities vs*.* time from **a** to **d**

The as-synthesized CsSn_0.5_Ge_0.5_I_3_ perovskite powders when exposed to ambient atmosphere (25 °C, 80% RH) for 24 h remain black and maintain their phase purity (Supplementary Fig. 1d). Moreover, it appears that the CsSn_*x*_Ge_1–*x*_I_3_ perovskite can become quite stable when the *x* value is in the range of 0.25–0.75 (Supplementary Fig. 8). In contrast, reference pure CsSnI_3_ and CsGeI_3_ powders turn into yellow non-perovskite phases (Supplementary Fig. 1f and 1e). These results demonstrate clearly the superior air stability of the alloy CsSn_0.5_Ge_0.5_I_3_ perovskite over its pure components. The CsSn_0.5_Ge_0.5_I_3_ perovskite thin films subjected to continuous 1-sun illumination in ambient atmosphere (~45 °C, 80% RH) for up to 72 h are also found to be highly stable. Figure 3a presents XRD patterns from CsSn_0.5_Ge_0.5_I_3_ perovskite thin films exposed for 0, 24, 48, and 72 h showing negligible change. The corresponding relative intensities of the main XRD peak are plotted in Fig. 3e. Comparative experiments on thin films of the popular halide perovskites (CsSnI_3_, CsPbI_3_, and MAPbI_3_) show complete degradation after 24, 48, and 72 h, respectively (Fig. 3b–d). Supplementary Fig. 9 presents conductive AFM (c-AFM) images of the CsSn_0.5_Ge_0.5_I_3_ perovskite thin film samples exposed to ambient atmosphere for 0, 24, and 48 h, where the microstructure and conductivity appear unchanged at the nanoscale.

### Physical properties of CsSn_0.5_Ge_0.5_I_3_ perovskite thin films

The time-resolved PL (TRPL) spectroscopy data from CsSn_0.5_Ge_0.5_I_3_ perovskite thin films (on glass substrates) in Supplementary Fig. 10a reveal a promising lifetime of 10.92 ns, compared with only 510 ps for the reference CsSnI_3_ thin film. Supplementary Fig. 10b shows TRPL results for CsSn_0.5_Ge_0.5_I_3_ perovskite thin films coated with either phenyl-C_61_-butyric acid methyl ester:C_60_ (PCBM:C_60_) or spiro-OMeTAD as electron- or hole-quenching layers, respectively. Based on the PL decay dynamics, the photogenerated carrier diffusion coefficients are estimated at 0.85 cm^2^ s^−1^ and 0.39 cm^2^ s^−1^ for electrons and holes^14,32^, respectively. These correspond to diffusion lengths of 963 nm and 653 nm for electrons and holes, respectively, which are sufficiently long for planar thin-film solar cells.

The carrier mobilities in the CsSn_0.5_Ge_0.5_I_3_ perovskite thin films were determined by the space-charge-limited-current (SCLC) method using symmetric capacitor-like devices shown schematically in Supplementary Fig. 11a and 11b insets. For determining the electron mobility (*μ*_e_) and the hole mobility (*μ*_h_) in the dark, the Ga/PCBM:C_60_/CsSn_0.5_Ge_0.5_I_3_/PCBM:C_60_/Ga and the Au/spiro-OMeTAD/CsSn_0.5_Ge_0.5_I_3_/spiro-OMeTAD/Au device structures, respectively, were used, in conjunction with the following equation:^33^

1 $$J_{{\mathrm{SCL}}} = \frac{{9\varepsilon \varepsilon _0\mu V^2}}{{8L^3}}$$

Here, *J*_SCL_ is the measured current density, *L* is the film thickness (=1 μm), *μ* is the carrier mobility, *ε* is the dielectric constant (=28), and *ε*_o_ is the permittivity of free space. The *μ*_e_ and *μ*_h_ are estimated at 974 cm^2^ V^−1^ s^−1^ and 213 cm^2^ V^−1^ s^−1^, respectively, where the latter is consistent with the *μ*_h_ value of 298 cm^2^ V^−1^ s^−1^ determined using the Hall-effect measurements. The electron- and hole-trap densities (*n*_t_) were calculated using the following equation:^34,35^

2 $$n_{\mathrm{t}} = \frac{{V_{{\mathrm{TFL}}}2\varepsilon \varepsilon _0}}{{eL^2}}$$

where *V*_TFL_ is the trap-filled limit (TFL) voltage from the plots in Supplementary Fig. 11a and 11b; the electron- and hole-trap densities are estimated to be both as low as about 10^16^ cm^–3^. All of the above results indicate that the properties of CsSn_0.5_Ge_0.5_I_3_ perovskite thin films are highly suitable for thin-film planar solar cells.

### Native-oxide-passivated CsSn_0.5_Ge_0.5_I_3_ perovskite solar cells

**Fig. 4:**
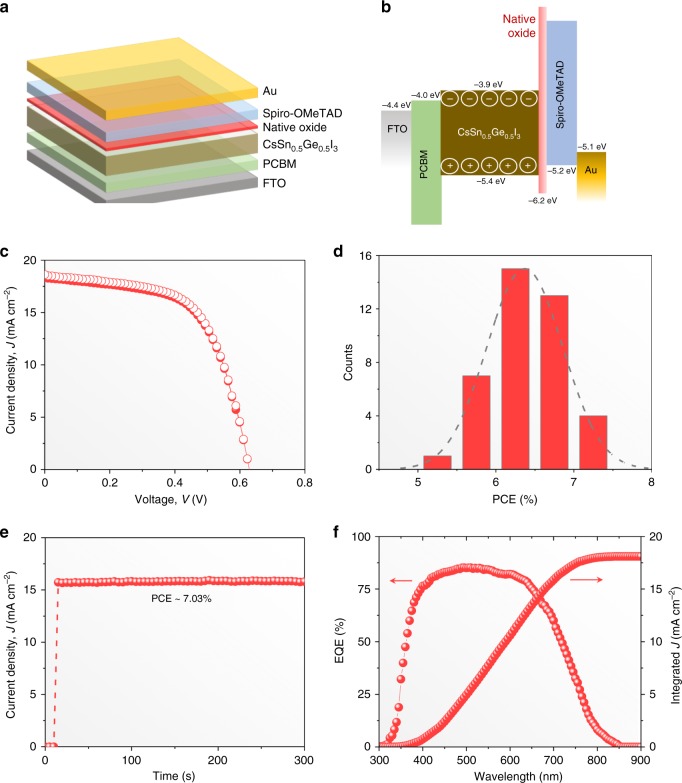
Device architecture and performance of CsSn_0.5_Ge_0.5_I_3_ thin-film PSCs. **a** Schematic illustration showing the planar PSC device structure used here. **b** Corresponding energy-level diagram. **c** *J–V* responses of the “champion” PSC device. **d** PCE statistics. **e** Stabilized power output and **f** EQE spectrum of the “champion” PSC device

The PV performance of CsSn_0.5_Ge_0.5_I_3_ perovskite thin films with the native-oxide layer is evaluated by incorporating them into PSC devices of the architecture shown in Fig. 4a. The CsSn_0.5_Ge_0.5_I_3_ perovskite thin film (about 200-nm thickness) is sandwiched between PCBM electron-transport layer (ETL) and spiro-OMeTAD hole-transport layer (HTL), with the thin native-oxide layer serving as a wide-bandgap interfacial layer between the CsSn_0.5_Ge_0.5_I_3_ perovskite thin film and the HTL. FTO and Au are used as the conducting electrodes. Figure 4b shows the energy-level diagrams for this device architecture. The VBM and the apparent bandgap of the amorphous native oxide were determined using the ultraviolet photoemission spectroscopy (UPS) results presented in Supplementary Fig. 12. The VBM of the CsSn_0.5_Ge_0.5_I_3_ thin film is determined by performing UPS measurement after the removal of the native-oxide layer using Ar sputtering (Supplementary Fig. 13). Figure 4c plots the current density (*J*)–voltage (*V*) responses of the “champion” PSC in reverse and forward scans, showing negligible hysteresis. High overall PCE of 7.11% is achieved with open-circuit voltage (*V*_OC_) of 0.63 V, short-circuit current density (*J*_SC_) of 18.61 mA cm^–2^, and fill factor (FF) of 0.606. The *J*_SC_ value is consistent with the integrated *J* of 18.1 mA cm^–2^ from the external quantum efficiency (EQE) spectrum in Fig. 4f. The PCE output at the maximum power point (0.45 V) shows 7.03% in Fig. 4e, which is very close to the extracted PCE from *J–V* curves. The *V*_OC_ reported here is much higher than that in other reported PSCs based on all-inorganic Sn-based halide perovskites^26,36^, and it appears to be stable (Supplementary Fig. 14). The evaluation of PV performance of about 40 PSC devices shows good reproducibility (Fig. 4d), with average PCE of 6.48%. More detailed performance statistics are included in Supplementary Fig. 15, and the device performance parameters are included in Supplementary Table 1.

In order to study the effect of the native-oxide layer on the PSC performance, control PSCs based on CsSn_0.5_Ge_0.5_I_3_ perovskite thin films, without the native oxide, were also fabricated. This was accomplished by performing all the processing steps inside a N_2_-filled glovebox (O_2_ and H_2_O levels below 0.1 ppm). Note that the processing of the PSCs described earlier was performed outside of the glovebox. The control PSC shows much lower *V*_OC_ of 0.48 V (reverse scan) and PCE of 3.72% (Supplementary Fig. 16a). By comparison, a PSC with the native-oxide layer shows significantly higher *V*_OC_ of 0.62 V (reverse scan) and PCE of 6.52% (Supplementary Fig. 16b). Also, a control PSC based on CsSnI_3_ perovskite was fabricated using the same evaporation method, showing low PCE of 1.7% (Supplementary Fig. 17). This clearly demonstrates the advantage of the mixed CsSn_0.5_Ge_0.5_I_3_ perovskite composition, and the beneficial effect of the native oxide. The corresponding dark *J–V* responses are also plotted in Supplementary Figs. 16a and 16b, showing lower current leakage when the native-oxide layer is present.

### Long-term device stability

**Fig. 5:**
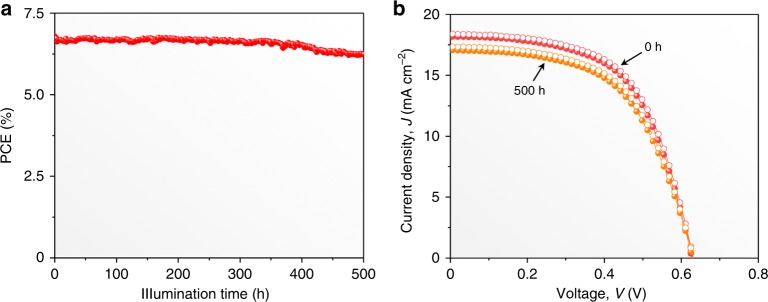
Device stability of CsSn_0.5_Ge_0.5_I_3_ thin-film PSCs. **a** PCE evolution of a typical unencapsulated PSC in continuous operation under 1-sun illumination at 45 °C in N_2_ atmosphere. **b** Initial *J–V* curves (reverse and forward scans) of the PSC and ones at the 500-h mark during continuous operation

A typical unencapsulated CsSn_0.5_Ge_0.5_I_3_ thin-film PSC with 6.79% PCE (Fig. 5b) was chosen for stability testing where it was operated continuously under continuous 1-sun illumination in N_2_ atmosphere (45 °C). The continuous monitoring of the PV performance shows that the PCE has degraded to only 6.23% (92% of the initial PCE) after 500 h of continuous operation (Fig. 5a, b). Figure 5b also shows that there is no degradation in the *V*_OC_. This demonstrates clearly the excellent operational stability of PSCs based on CsSn_0.5_Ge_0.5_I_3_ perovskite thin films with the native-oxide layer. Regarding the stability in air, we have periodically measured the *J–V* performance of a CsSn_0.5_Ge_0.5_I_3_-based PSC that was stored under 1-sun illumination. As shown in Supplementary Fig. 18, after 100-h storage, the PSC promisingly maintains 91% of the initial PCE. Overall, to the best of our knowledge, such stability is the highest of all lead-free perovskite PSCs reported so far. Note that, while it is practically challenging to perform a direct performance comparison between the CsSn_0.5_Ge_0.5_I_3_-based and Pb-based PSCs with the same device configuration, the stability of the CsSn_0.5_Ge_0.5_I_3_-based PSCs is reasonably comparable to that of Pb-based PSCs^5,37^.

## Discussion

We have shown that thin-film PSCs based on all-inorganic, Pb-free perovskites of composition CsSn_0.5_Ge_0.5_I_3_ show superior performance and exceptional stability. The air stability of CsSn_0.5_Ge_0.5_I_3_ perovskite over its pure counterparts CsSnI_3_ and CsGeI_3_ is attributed to the formation of the passivating, stable native-oxide layer on CsSn_0.5_Ge_0.5_I_3_ perovskite surfaces when exposed to air. As gleaned from the XPS results, the native oxide on CsSn_0.5_Ge_0.5_I_3_ perovskite thin films comprises GeO_2 _ doped with a small portion of Sn. It may be that Sn doping suppresses the formation of those volatile, unstable Ge suboxides during surface passivation in air^38,39^. It is likely that the Sn-doping also enhances the moisture stability of GeO_2 _itself, making the passivation layer stable in air. Further studies will be performed to understanding the details of Sn-doping effects. Also, as shown in Supplementary Fig. 7, the entropy-of-mixing contributes to 0.03 eV (1.2*k*_B_*T* at room temperature, *k*_B_ is the Boltzmann constant and *T* is 300 K) in reduced free energy in the CsSn_0.5_Ge_0.5_I_3_ perovskite, adding to its thermodynamic stability.

It is confirmed that the presence of the native-oxide layer on CsSn_0.5_Ge_0.5_I_3_ perovskite thin films is also important for the enhanced PV performance (Supplementary Fig. 16). This can be attributed to the suppression of recombination of photocarriers at the interface between the CsSn_0.5_Ge_0.5_I_3_ perovskite and the HTL where the native oxide resides. Also, the dark *J–V* results in Supplementary Figure 16 indicate improved hole transport across that interface and enhanced shunt resistance, resulting in the enhanced PV performance. The remarkable operational stability of the PV performance is again attributed to the passivating nature of the Sn-containing GeO_2_ native-oxide layer that protects the thin-film surfaces and interfaces, and also the enhanced intrinsic or thermodynamic stability of the CsSn_0.5_Ge_0.5_I_3_ perovskite.

The CsSn_0.5_Ge_0.5_I_3_ perovskite composition studied here demonstrates the “proof-of-concept” of the efficacy of the native-oxide approach in stabilizing materials and devices. Currently, we have relied on the native oxide that forms naturally upon exposure to air, which may not be optimum. Thus, an investigation where the native oxide is tailored via controlled heat treatments (temperature, time, and oxygen partial pressure) is likely to be a fruitful research direction. Tuning the electrical and chemical properties of native oxide, and also exploring other ETL, HTL, and electrode materials with energy levels better suited for the cesium tin-germanium triiodide  alloy perovskites, may result in PSCs with further enhanced performance. Finally, the native-oxide approach demonstrated for achieving enhanced PV performance and operational stability could be extended rationally to other halide-perovskite compositions.

## Methods

### Synthesis of CsSn_0.5_Ge_0.5_I_3_ raw powders and films

All raw materials were purchased from Sigma Aldrich (USA) and used without further purification. CsSn_0.5_Ge_0.5_I_3_ perovskite powder was synthesized by solid-state reaction between mixed solid powder precursors CsI:SnI_2_:GeI_2_ (2:1:1 molar ratio) carried out in evacuated Pyrex tubes. The tubes were evacuated to 10^–3^ Torr pressure for at least 6 h before sealing them using an oxy-methane torch under vacuum. The evacuated tubes with the powder mixture were then placed in a tube furnace and heated to 450 °C and held for 72 h, followed by slow cooling at a rate of 20 °C h^–1^ to room temperature. The as-synthesized CsSn_0.5_Ge_0.5_I_3_ perovskite powder was evaporated to deposit thin films of the same composition on various substrates using a thermal evaporator (Edwards/306A; UK). The deposition was carried out at 10^–5^ Torr under low current settings of 36 to 42 mA.

### Materials characterization

XRD of the CsSn_0.5_Ge_0.5_I_3_ perovskite powders and thin films (on glass substrate) was performed using a high-resolution diffractometer (D8 Advance, Bruker; Germany) with CuK_α_ radiation. UV-vis spectra were obtained using a spectrophotometer (UV-2600, Shimadzu; Japan). The microstructures of the thin films were observed using a SEM (LEO 1530VP, Carl Zeiss; Germany). Hall-effect measurements were conducted on a device with four-Ga electrode system (2400 SourceMeter, Keithley; USA). The majority carrier was determined to be holes based on the Hall-voltage sign. An XPS and UPS system (5600, PHI; USA) was used to acquire both angle-dependent XPS and UPS spectra. The analysis chamber base pressure was <10^–9^ Torr prior to analysis. The instrument utilized a monochromated Al-K_α_ source for X-ray radiation at 1486.7 eV, and a UVS 40A2 (PREVAC, Poland) UV source and UV40A power supply provided by He 1_α_ for UPS at 21.22 eV. Chamber pressure for UPS was maintained at less than 3 × 10^–8^ Torr. XPS data were collected at different incidence angles. In some cases, in situ Ar sputtering (15 s) was used to etch away the surface layer. XPS mapping was performed on another XPS system (AXIS ULTRA HAS, Kratos Analytical; UK). The determination for the ratio of valence states and atom ratios was performed by Casa XPS (2.3.19) and Origin. The measurement conditions were 150 W of applied power to the X-ray source under ultrahigh vacuum (10^–9^ Torr). The images were collected using field of view (200 × 200 μm^2^) with the high-resolution imaging Iris (<3 μm). The binding energies of the elements of interest for generating the maps were first defined by acquiring the XPS spectra. Acquisition time for each elemental mapping was 2 min. The SCLC *I–V* curves were measured using the 2400 SourceMeter (Keithley; USA). Thicker films (1 μm) are used here to eliminate current leakage. However, it should be noted that thicker films may amplify the mobility and lower the trap density. The refractive indices of the CsSn_0.5_Ge_0.5_I_3_ perovskite thin films were measured using an ellipsometer (M-7000, J.A. Woollam; USA) at an incidence angle of 75°. The dielectric constant (*ε*) was accordingly calculated from electrical capacity test. The steady-state and time-resolved PL spectra were recorded using a spectrophotometer (Varian Cary Eclipse Fluorescence, Agilent; USA) operated at 395-nm excitation. The decay rate and lifetime were determined using the three-parameter decay function fitting method.

### Device fabrication and testing

Patterned FTO-coated glass (Hartford Glass Co.; USA) substrates were cleaned successively with detergent solution, acetone, and isopropanol, and they were UV-ozone treated for 20 min prior to the deposition of the other layers. A less than 20-nm PCBM layer using a 20 mg mL^–1^ solution of PCBM in 1,2-dichlorobenzene was spin-coated (3000 rpm, 60 s). CsSn_0.5_Ge_0.5_I_3_ perovskite thin films were then deposited using the method described above. The HTL solution was prepared by dissolving 91 mg of spiro-OMeTAD (Merck, Germany) with additives in 1 mL of chlorobenzene. The additives were 21 µL of Li-bis(trifluoromethanesulfonyl) imide from the stock solution (520 mg in 1 mL of acetonitrile), 16 µL of FK209 (tris(2-(1H-pyrazol-1-yl)-4-tert-butylpyridine)-cobalt(III) tris(bis(trifluoromethylsulfonyl) imide) (375 mg in 1 mL of acetonitrile), and 36 µL of 4-tertbutylpyridine. The HTL solution was spin-coated (4000 rpm, 20 s), followed by the deposition of the 80-nm-thick Au electrode by thermal evaporation. Control PSCs were also fabricated, where all the deposition steps were conducted in a N_2_-filled glovebox (O_2_ and H_2_O levels <0.1 ppm) to prevent the formation of the native oxide on the surface of the CsSn_0.5_Ge_0.5_I_3_ perovskite thin films. The *J–V* characteristics of all the PSCs were measured using the 2400 SourceMeter under simulated 1-sun AM1.5G 100 mA∙cm^–2^ intensity (Sol3A Class AAA, Oriel, Newport; USA) in ambient atmosphere, using both reverse (from *V*_OC_ to *J*_SC_) and forward (from *J*_SC_ to *V*_OC_) scans with a step size of 0.015 V and a delay time of 100 ms. The maximum-power output stability of PSCs was measured by monitoring the *J* output at the maximum power-point bias (deduced from the reverse-scan *J–V* curves) using the 2400 SourceMeter. A typical active area of 0.1 cm^2^ was defined using a non-reflective mask for the *J–V* measurements. The stable output PCE was calculated using the following relation: PCE=*J* (mA cm^–2^)×*V* (V)/100 (mA V cm^–2^). A shutter was used to control the 1-sun illumination on the PSC. The EQE spectra were obtained using a quantum efficiency measurement system (IQE 200B, Oriel; USA). For long-term device stability test, the PSCs were placed in a holder with a transparent-glass cover and a continuous flow of nitrogen gas. The current/PCE outputs of PSCs at the maximum-power point were monitored under continuous 1-sun-intensity (white-LED) illumination.

### DFT calculations

All first-principles computations were performed based on density-functional theory (DFT) methods as implemented in the Vienna ab initio simulation package (VASP 5.4). An energy cutoff of 520 eV was employed, and the atomic positions were optimized using PBEsol functional until the maximum force on each atom was less than 0.02 eV∙Å^–1^. The ion cores were described by using the projector-augmented wave (PAW) method. A 4 × 4 × 4 *k*-point grid was used for the mixed perovskites. The simulation cells of mixed perovskites with different stoichiometry containing eight units were constructed by replacing the corresponding Sn(II) or Ge(II). For each composition, the geometry and cell length were then fully relaxed. This justifies the estimation of the entropy-of-mixing contribution to the free energy by using the analytical formula for ideal alloys: $T{\mathrm{\Delta }}S = - k_{\mathrm{B}}T\left[ {x{\mathrm{ln}}x + \left( {1 - x} \right)\ln \left( {1 - x} \right)} \right]$. The mixed energy contributions to the free energy can also be estimated based on the formula: ${\mathrm{\Delta }}E_{{\mathrm{mixed}}} = E_{{\mathrm{CsSn}}_x{\mathrm{Ge}}_{(1 - x)}{\mathrm{I}}_3} - (xE_{{\mathrm{CsSnI}}_3} + (1 - x)E_{{\mathrm{CsGeI}}_3})$.

## Supplementary information

Supplementary Information

Solar Cells Reporting Summary

## Data Availability

The authors declare that the data related to this study are available upon reasonable request.
