# Supplementary material for: Highly stable and efficient all-inorganic lead-free perovskite solar cells with native-oxide passivation
Source: Nat Commun. 2019 Jan 3;10:16. doi: 10.1038/s41467-018-07951-y (PMC6318336; doi:10.1038/s41467-018-07951-y)
Supplement: Supplementary file 1 — Supplementary Information [file 41467_2018_7951_MOESM1_ESM.pdf]

Supplementary Information

Highly Stable and Efficient All-Inorganic Lead-Free Perovskite Solar Cells with Native-Oxide Passivation

Chen et al

Supplementary Information

Highly Stable and Efficient All-Inorganic Lead-Free Perovskite Solar Cells with Native-Oxide Passivation

Min Chen,[1] Ming-Gang Ju,[2] Hector F. Garces,[1] Alexander D Carl,[3] Katsuya Ono,[4] Zafer Hawash,[4] Yi Zhang,[1] Tianyi Shen,[1] Yabing Qi,[4] Ronald L. Grimm,[3] Domenico Pacifici,[1] Xiao Cheng Zeng,[2] Yuanyuan Zhou,[1,*] Nitin P. Padture[1,*]

[1] School of Engineering, Brown University, Providence, Rhode Island 02912, United States

[2] Department of Chemistry, University of Nebraska−Lincoln, Lincoln, Nebraska 68588, United States

[3] Department of Chemistry and Biochemistry, Worcester Polytechnic Institute, Worcester, MA 01609, United States

[4] Energy Materials and Surface Sciences Unit, Okinawa Institute of Science and Technology Graduate University, Okinawa 904-0495, Japan

[*]Contact: yuanyuan_zhou@brown.edu (Y.Z.); nitin_padture@brown.edu (N.P.P.);

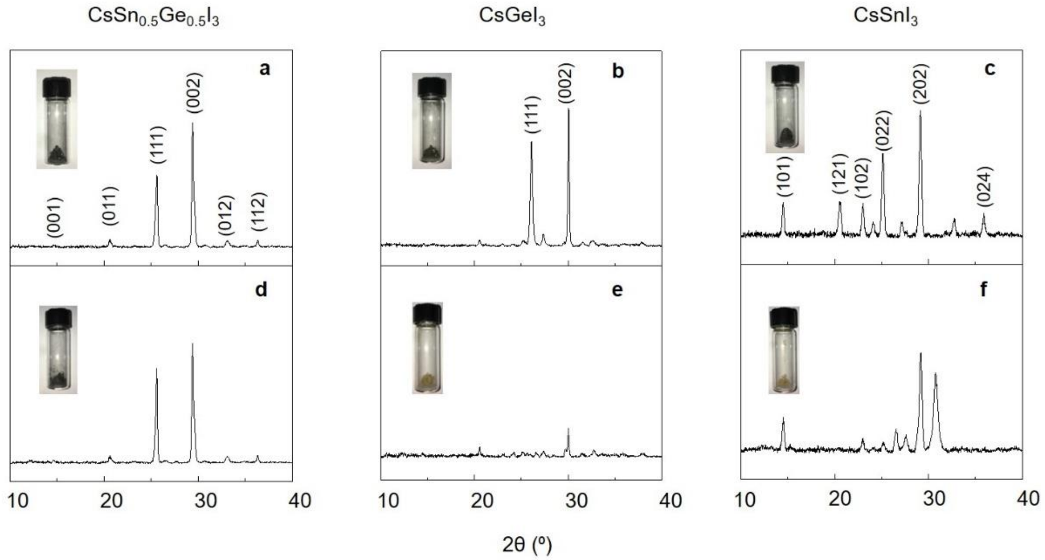

Supplementary Figure 1. Relative stability $CsSn_{0.5}Ge_{0.5}I_3$, $CsGeI_3$ and $CsSnI_3$. (a, b, c) XRD patterns of as-synthesized $CsSn_{0.5}Ge_{0.5}I_3$, $CsGeI_3$ and $CsSnI_3$ powders, respectively. (d, e, f) XRD patterns of $CsSn_{0.5}Ge_{0.5}I_3$, $CsGeI_3$, and $CsSnI_3$ powders after 24-h exposure to air (25 ˚C, 80% RH), respectively. Insets: photograph of the respective powders.

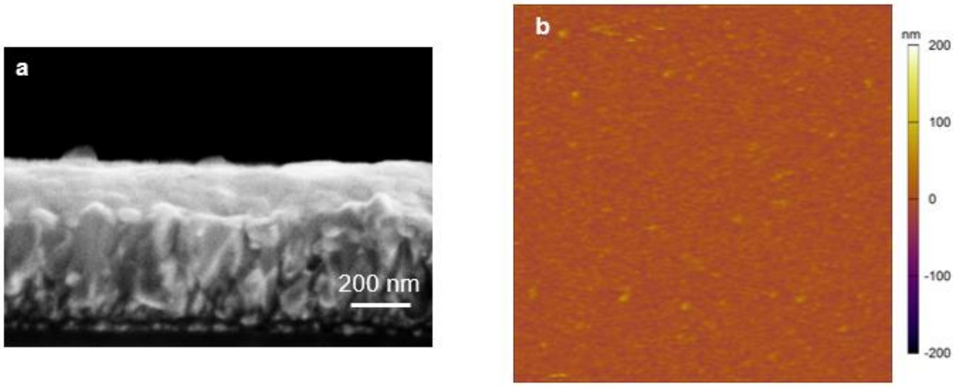

Supplementary Figure 2. Thin film morphology characterization. (a) Cross-sectional SEM of the $CsSn_{0.5}Ge_{0.5}I_3$ perovskite thin film. (b) Top-surface AFM image of the $CsSn_{0.5}Ge_{0.5}I_3$ perovskite thin film over a 50×50-μm^2 area.

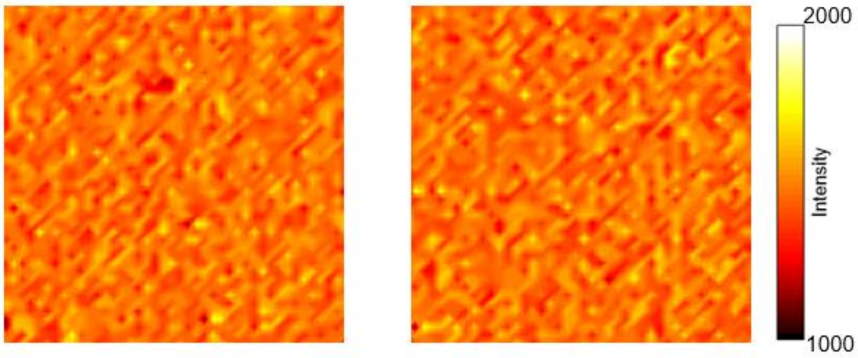

Supplementary Figure 3. PL mapping. PL maps over 50×50-μm^2 areas from two different regions in the same $CsSn_{0.5}Ge_{0.5}I_3$ perovskite thin film.

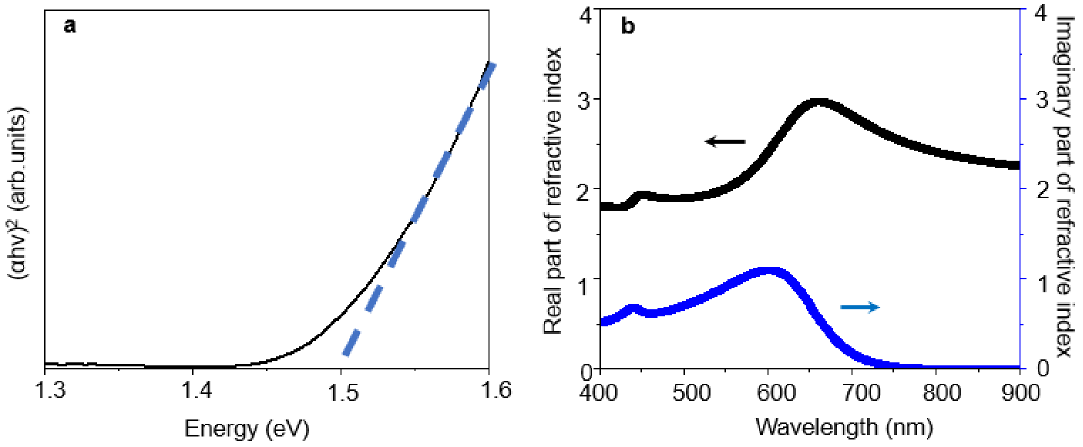

Supplementary Figure 4. Optical characterization. (a) Tauc plot of the $CsSn_{0.5}Ge_{0.5}I_3$ perovskite thin film. (b) Refractive index of the $CsSn_{0.5}Ge_{0.5}I_3$ perovskite thin film fitted from the ellipsometry data.

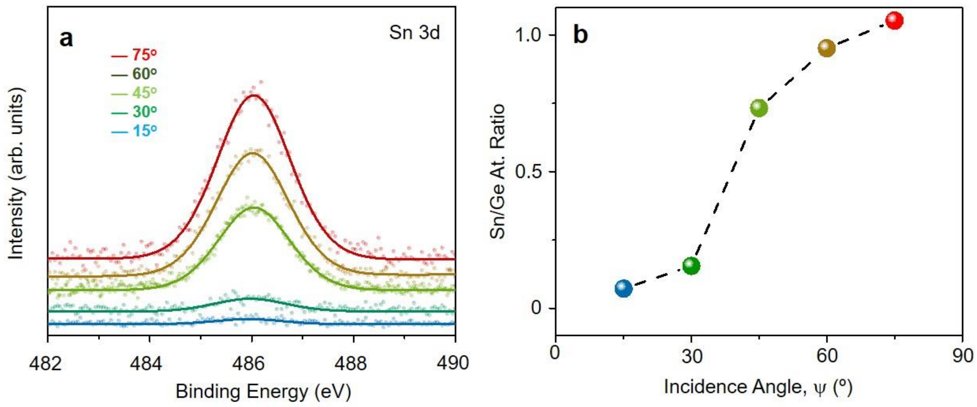

Supplementary Figure 5. XPS characterization. (a) Sn 3d XPS spectra, at different incidence angles, from $CsSn_{0.5}Ge_{0.5}I_3$ perovskite thin film that has been exposed to air. (b) Sn:Ge atomic ratio extracted from the Ge 3d and Sn 3d XPS data at different incidence angles.

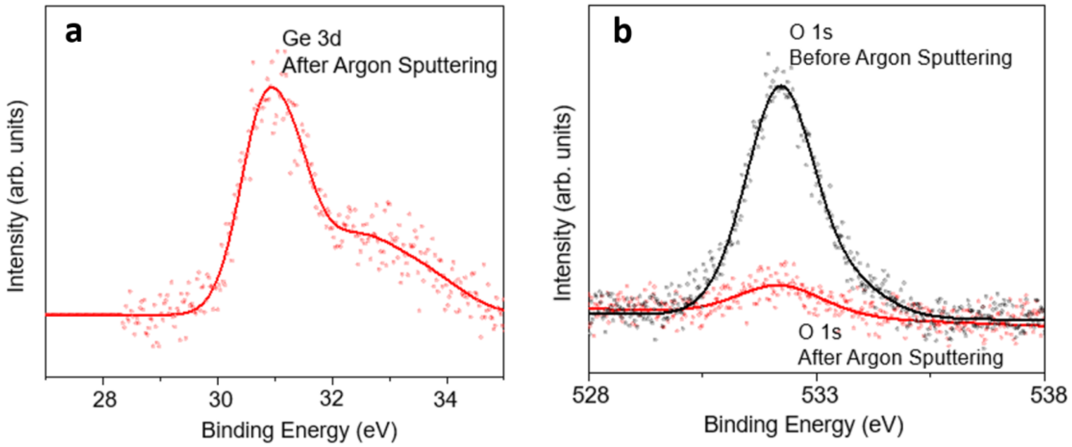

Supplementary Figure 6. XPS characterization. (a) XPS spectrum (Ge 3d) of $CsSn_{0.5}Ge_{0.5}I_3$ perovskite thin film (incidence angle: 15˚) after 15-s Ar-sputtering. (b) XPS spectra (O 1s) of $CsSn_{0.5}Ge_{0.5}I_3$ perovskite thin film (incidence angle: 15˚) before (black) and after (red) 15-s Ar-sputtering.

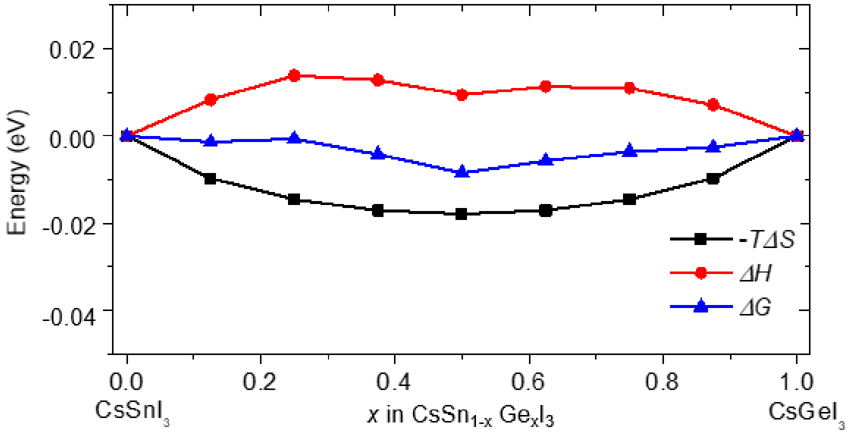

Supplementary Figure 7. Energy calculations. Calculated enthalpy, entropy, and Gibbs free energy at 300 K for $CsSn_{1-x}Ge_xI_3$ as a function of x. For simplicity, the $CsSn_{0.5}Ge_{0.5}I_3$ phase was assigned to have the space group of $\boldsymbol{R3m}$.

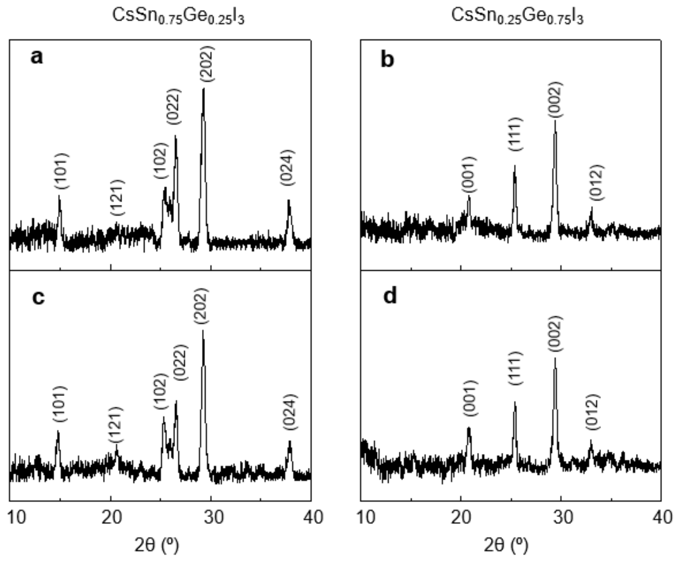

Supplementary Figure 8. Relative stability of $CsSn_{0.75}Ge_{0.25}I_3$ and $CsSn_{0.25}Ge_{0.75}I_3$. (a, b) XRD patterns of as-synthesized $CsSn_{0.75}Ge_{0.25}I_3$ and $CsSn_{0.25}Ge_{0.75}I_3$ powders, respectively. (c, d) XRD patterns of $CsSn_{0.75}Ge_{0.25}I_3$ and $CsSn_{0.25}Ge_{0.75}I_3$ powders after 24-h exposure to air (25 ˚C, 80% RH), respectively.

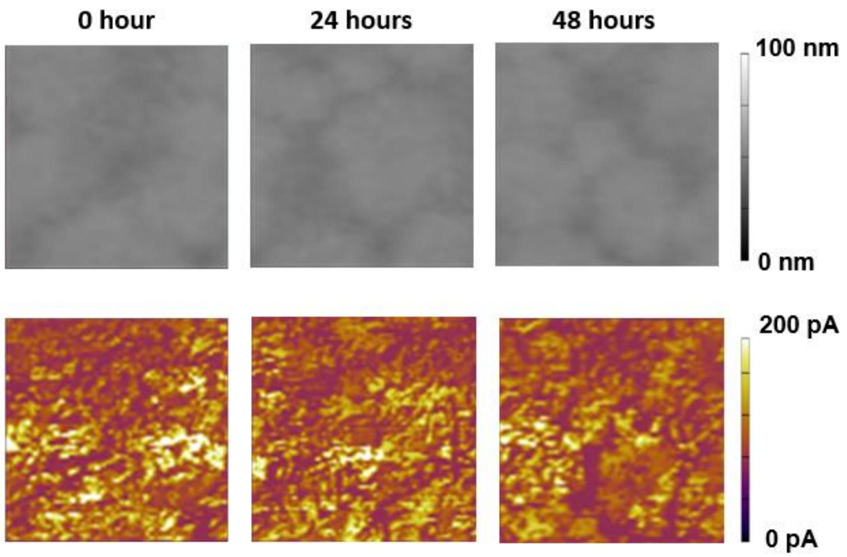

Supplementary Figure 9. AFM and *c*-AFM mapping. AFM topography (top) and corresponding *c*-AFM (bottom) maps over a 5×5-μm^2 area of the $CsSn_{0.5}Ge_{0.5}I_3$ perovskite thin film exposed to air for 0, 24 h, and 48 h.

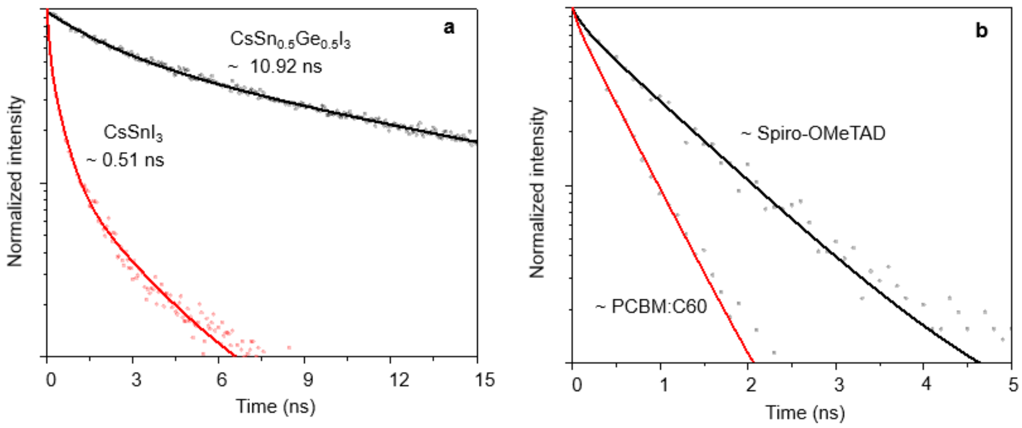

Supplementary Figure 10. TRPL spectroscopy. (a) TRPL results from the $CsSn_{0.5}Ge_{0.5}I_3$ and the $CsSnI_3$ perovskite thin film (thickness ~200 nm on glass substrates). (b) PL decay dynamics of the $CsSn_{0.5}Ge_{0.5}I_3$ perovskite thin films coated with an electron (PCBM:C_{60}) or a hole quenching (Spiro-OMeTAD) layer.

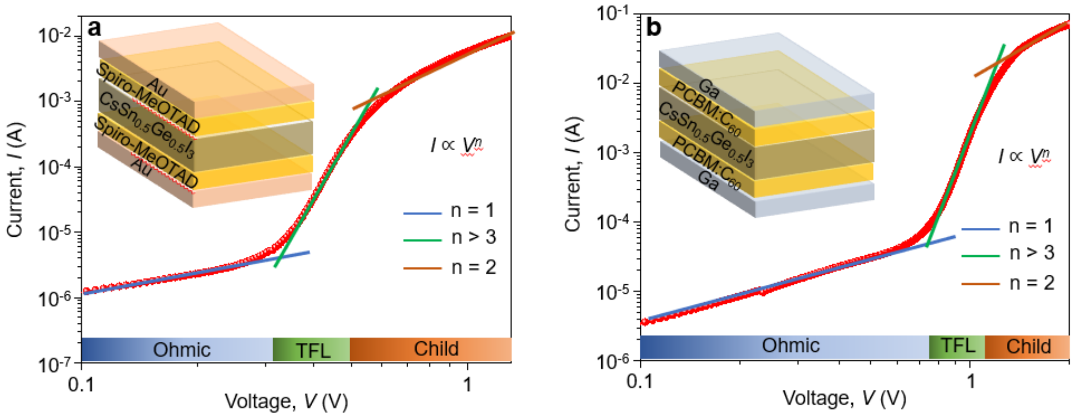

Supplementary Figure 11. Film electrical properties. *I*-*V* characteristics of $CsSn_{0.5}Ge_{0.5}I_3$ perovskite thin films for (a) hole-only transport (b) electron-only transport. Insets: the respective device architectures used.

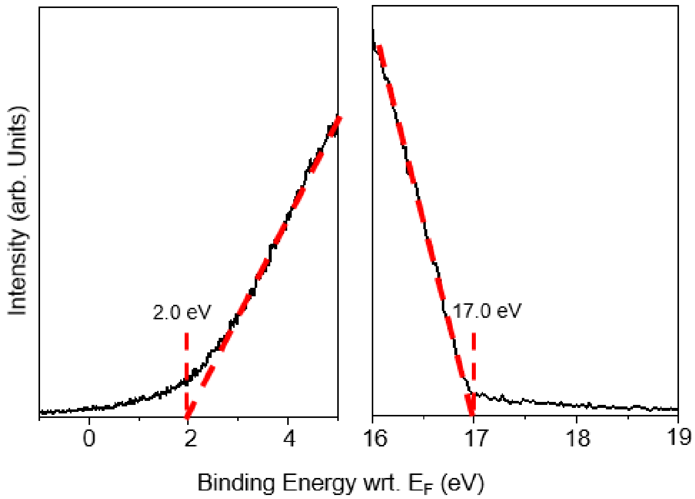

Supplementary Figure 12. UPS characterization. UPS data from the native oxide on the $CsSn_{0.5}Ge_{0.5}I_3$ perovskite thin film.

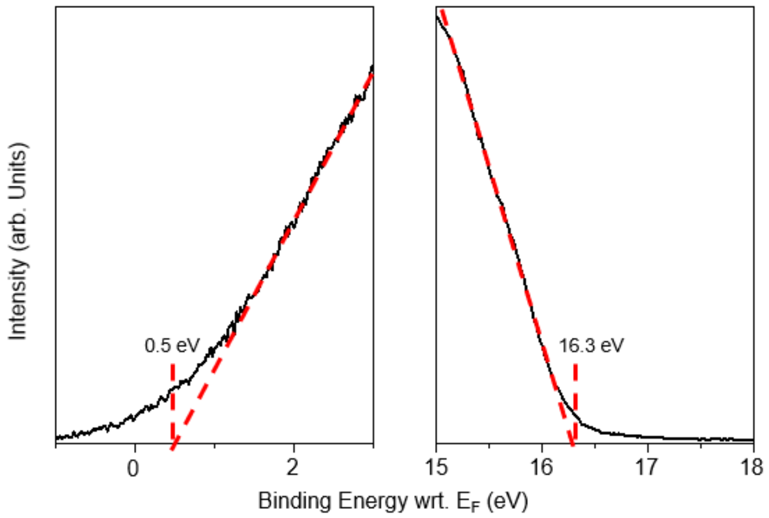

Supplementary Figure 13. UPS characterization. UPS data from the $CsSn_{0.5}Ge_{0.5}I_3$ perovskite thin film after 15-s Ar-sputtering.

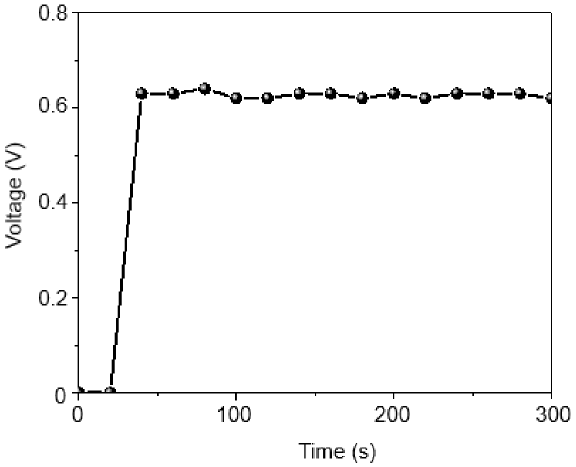

Supplementary Figure 14. V_{OC} stability. The stability of the V_{OC} of the PSC based on the $CsSn_{0.5}Ge_{0.5}I_3$ perovskite thin film. The V_{OC} has been determined every other 20 seconds.

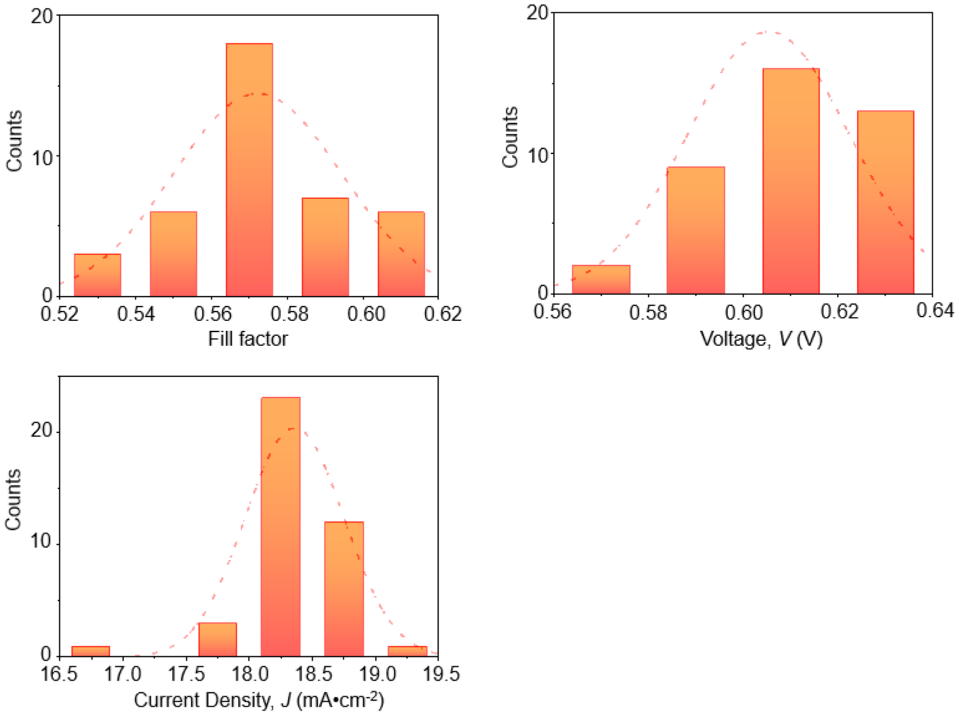

Supplementary Figure 15. Performance statistics. Detailed statistics of J_{SC}, V_{OC} and FF from 40 $CsSn_{0.5}Ge_{0.5}I_3$-based PSCs.

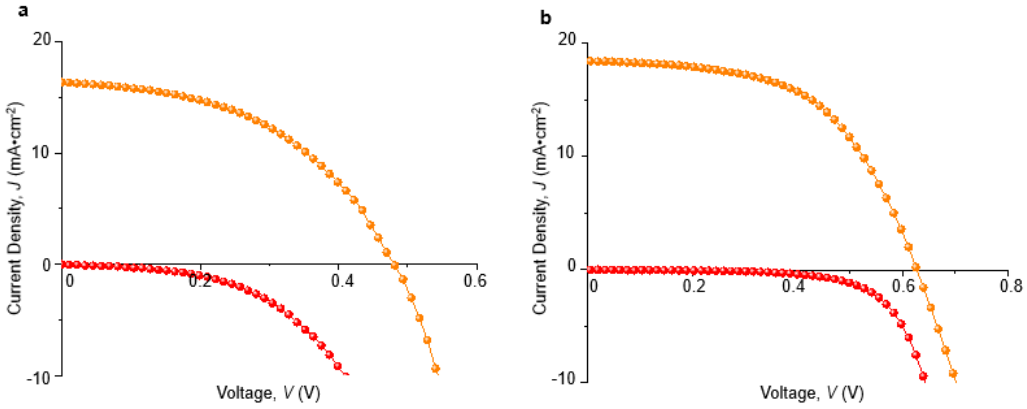

Supplementary Figure 16. Performance of PSCs based on $CsSn_{0.5}Ge_{0.5}I_3$ perovskite thin films. *J-V* responses (reverse scan) under 1-sun and in 'dark': (a) PSC without native oxide fabricated inside a N_2-filled glovebox (O_2 and H_2O levels <0.1ppm) and (b) PSC with native oxide fabricated in ambient atmosphere.

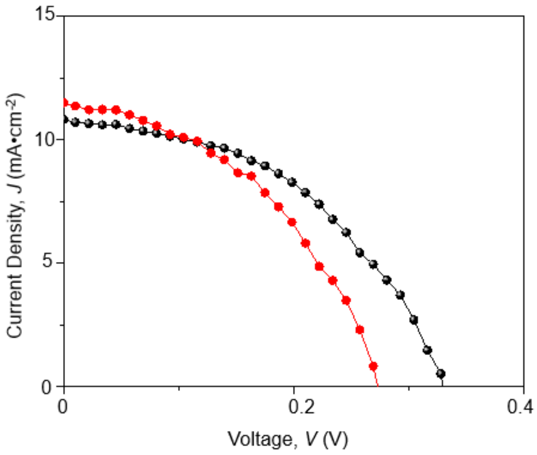

Supplementary Figure 17. Performance of $CsSnI_3$-based PSCs. *J*-*V* curves of $CsSnI_3$-based PSCs fabricated using the similar single-source evaporation method that is used for $CsSn_{0.5}Ge_{0.5}I_3$ in this work (red: forward scan, black: reverse scan).

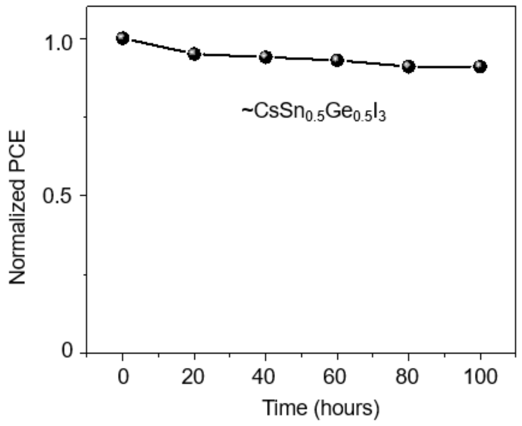

Supplementary Figure 18. Air-stability. PCE Stability of $CsSn_{0.5}Ge_{0.5}I_3$-based PSCs (unencapsulated) under continuous light illumination in the ambient air.

Supplementary Table 1. *J-V* parameters of the 40 PSC devices used for the statistics.

Device No.	V_{OC} (V)	FF	J_{SC} (mA•cm^{-2})	PCE (%)
1	0.59	0.562	18.457	6.119
2	0.59	0.556	18.625	6.11
3	0.59	0.544	18.351	5.89
4	0.59	0.544	18.164	5.83
5	0.58	0.544	17.653	5.57
6	0.58	0.536	17.884	5.559
7	0.57	0.536	18.198	5.56
8	0.57	0.536	16.758	5.119
9	0.63	0.613	18.41	7.11
10	0.63	0.613	18.255	7.049
11	0.63	0.608	18.379	7.039
12	0.63	0.608	18.353	7.029
13	0.63	0.608	18.248	6.99
14	0.62	0.605	18.261	6.85
15	0.62	0.595	18.181	6.707
16	0.62	0.592	18.526	6.8
17	0.62	0.592	18.472	6.78
18	0.59	0.562	18.456	6.119
19	0.61	0.568	18.615	6.449
20	0.61	0.568	18.327	6.35
21	0.61	0.566	18.334	6.33
22	0.6	0.566	18.404	6.249
23	0.6	0.566	18.345	6.229
24	0.6	0.566	18.345	6.229
25	0.6	0.566	18.256	6.199
26	0.6	0.566	18.021	6.12
27	0.6	0.566	18.021	6.12
28	0.62	0.586	18.661	6.78
29	0.62	0.586	18.661	6.78
30	0.62	0.583	18.757	6.779
31	0.61	0.576	19.296	6.78
32	0.61	0.576	18.869	6.63
33	0.61	0.576	18.641	6.55
34	0.61	0.573	18.71	6.54
35	0.61	0.569	18.669	6.48
36	0.61	0.568	18.673	6.47
37	0.6	0.566	18.346	6.229
38	0.58	0.544	18.16	5.73
39	0.58	0.544	17.97	5.669
40	0.62	0.588	18.597	6.779
